# Mosaic *TP53* Mutation on Tumour Development in Pigs: A Case Study

**DOI:** 10.1155/2023/7000858

**Published:** 2023-08-14

**Authors:** Chommanart Thongkittidilok, Maki Hirata, Qingyi Lin, Nanaka Torigoe, Bin Liu, Yoko Sato, Megumi Nagahara, Fuminori Tanihara, Takeshige Otoi

**Affiliations:** ^1^Bio-Innovation Research Center, Tokushima University, Tokushima, Japan; ^2^Faculty of Bioscience and Bioindustry, Tokushima University, Tokushima, Japan; ^3^Department of Veterinary Science, School of Biological Science, Tottori University, Tottori, Japan

## Abstract

Pigs rarely develop cancer; however, tumour protein p53 (*TP53*)-modified pigs may have an increased incidence of cancer. In this study, two pigs with mosaic mutations induced by gene editing were compared to determine the role of the wild-type *TP53* sequence in tumorigenesis and to speculate how amino acid changes in TP53 sequences are related to tumorigenesis. The pig without tumours had a wild-type *TP53* sequence and a 1-bp deletion in the *TP53* sequence that resulted in a premature stop codon. In contrast, the pig with nephroblastoma had 6- and 7-bp deletions in the *TP53* sequence, resulting in the absence of two amino acids and a premature stop codon, respectively. Our results indicated that *TP53* mutations with truncated amino acids may be related to tumour formation.

## 1. Introduction

Pigs are used as models for cancer research because of their similarities to humans. However, spontaneous cancer is uncommon in pigs, making it necessary to genetically modify them to establish a cancer model. The tumour protein p53 (*TP53*) gene induces apoptosis in response to DNA damage caused by cellular stress and is crucial for cancer development. Mutations in *TP53* are associated with almost all cancers and are linked to the amplification of oncogenes and deletion of tumour suppressor genes [1]. Cancers with wild-type *TP53* have different miRNA expression profiles than those with mutant *TP53*. In cancers with wild-type *TP53*, miRNAs associated with tumour suppression are enriched, promoting apoptosis and suppressing cell cycle progression. Conversely, in cancers with mutant *TP53*, miRNAs associated with oncogenic functions are enriched, thereby reducing apoptosis and promoting cell cycle progression. Cancers with mutant *TP53* also exhibit upregulation of proteins related to cell cycle progression and the DNA damage response [2].

In a previous study, pigs with *TP53* modifications targeting exon 3 and intron 4 exhibited various tumour phenotypes, including histiocytoma, osteosarcoma, and nephroblastoma [3]. This study compared the genotypic differences in tumorigenic and nontumorigenic phenotypes between two gene-edited pigs: one with a mosaic mutation and wild-type *TP53* sequence and the other with a mosaic mutation and no wild-type *TP53* sequence. We also performed Tracking Indels by DE composition (TIDE) analysis and protein alignment to investigate the relationship between the changes in amino acids in pigs with and without the wild-type *TP53* sequence.

## 2. Materials and Methods

Animal experiments were approved by the Institutional Animal Care and Use Committee of Tokushima University (approval number: T2019-11). Animal husbandry and anaesthesia/euthanasia were performed as previously described [3]. *TP53*-modified pigs were generated using the gene editing by electroporation of the Cas9 protein (GEEP) method using a single guide RNA (sgRNA) targeting exon 3 of TP53 (5′-GGTCTTCTGAGAAGGGACAA-3′), as previously described [3]. Exon 3 was targeted because a double-strand break (DSB) postindel mutation occurs in exon 3 before the final exon, with the expectation that the frameshift would result in a premature termination codon that knocks out gene expression [4]. In brief, Cas9 protein and sgRNA1 (Figure S1), used in the previous study [3], were introduced into in vitro-fertilized zygotes via electroporation. All electroporated zygotes (400 zygotes) were then transferred into the oviducts of one oestrous-synchronized recipient gilt. In the previous study, the blastocyst formation rate of electroporated zygotes was 18.1% [3], but the development of the zygotes used for this study was not examined because all electroporated zygotes were transferred. The recipient gave birth to eight mutant piglets, two (pigs A and B) of which were observed until a maximum age of 15 months.

Genomic DNA from ear biopsies (collected 1 d after birth) of piglets was analysed to determine the presence of *TP53* and the level of mosaicism, as described previously [5]. In brief, DNA was extracted from ear biopsies by boiling them in a 50 mM NaOH solution at 98°C for 10 min, followed by neutralization with 1 M Tris HCl (pH 8.0). After neutralization, the genomic regions flanking the sgRNA target sequences were amplified by two-step PCR using specific primers (5′-CGAACTGGCTGGATGAAAAT-3′ (forward) and 5′-CCAGGGTCCAAGGTCATAGA-3′ (reverse)) with overhang adapters and Index PCR Primers, following the manufacturer's instructions (Illumina, Hayward, CA, USA). PCR products were extracted and purified using a FastGene gel/PCR extraction kit (Nippon Genetics, Tokyo, Japan). Following gel purification, Illumina next-generation sequencing (NGS) techniques were used to sequence the amplicons using the MiSeq Reagent Kit v. 2 (250 cycles) (Illumina, San Diego, CA, USA).

Pig A with a mosaic mutation and wild-type *TP53* sequence and Pig B with a mosaic mutation and no wild-type *TP53* sequence were sacrificed at 13 and 15 months, respectively, under deep anaesthesia with isoflurane. Tissues from major organs, including the lungs, spleen, liver, heart, and kidneys, were carefully examined for the presence of tumours and subsequently collected for sequential and histological analyses. The indel frequency in the region of *TP53* in major organs was analysed with the TIDE bioinformatics package [6] using results from Sanger sequencing. The genomic regions flanking the sgRNA target sequences were amplified using PCR. The purified PCR product was directly sequenced by Sanger sequencing using a BigDye Terminator Cycle Sequencing Kit (version 3.1; Thermo Fisher Scientific, Waltham, MA, USA). The mutation rates were defined as the proportion of indel mutations. For histological analysis, tissues were preserved in 10% neutral-buffered formalin, manually embedded in paraffin, and stained with haematoxylin and eosin.

## 3. Results

Sequence analysis of the *TP53* genomic regions flanking the sgRNA target sequences demonstrated that pig A carried a mosaic genotype with 54.5% mutation (42% 1-bp deletion, 6.3% 6-bp insertion, and 6.2% 9-bp deletion) and 42.2% wild-type (Wt) in its genome, whereas pig B had no wild-type *TP53* sequence and carried a mosaic genotype with 97.3% mutations (49.7% 6-bp deletion and 47.7% 7-bp deletion) in exon 3 (Table 1). The total mutation frequency for each animal was not 100% because the error rate using the Illumina MiSeq platform was greater than 0.1% [7]. For an off-target analysis, we searched the whole genome sequence of the pig for potential off-target sites and analysed three sites for gRNA showing mismatches/gaps (Table S1). In a deep-sequencing analysis, we did not detect mutations at off-target sites in more than 99% of the amplicons in both pigs (Table S2). The remaining 1% was composed of a small number of amplicons (<0.1%) carrying different sequences.

**Figure 1:**
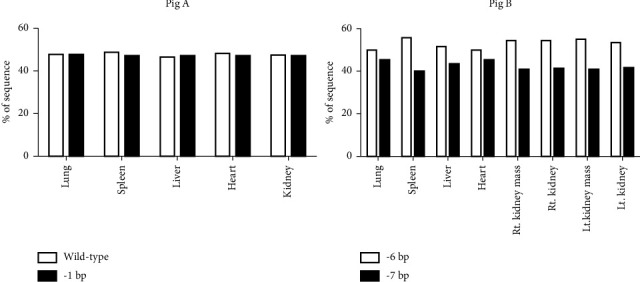
Frequency of the wild-type sequence (in pig A) and indel mutation in tissues of *TP53*-modified pigs (A and B) as determined by TIDE analysis.

In pig A, no tumours were detected macroscopically or histologically in any of the collected tissues. In all tissues collected, only the frequencies of wild-type and 1-bp deletion sequences were detected by TIDE analysis; however, a low frequency of mutations, including a 6-bp insertion and a 9-bp deletion, was not detected (Figure 1). The frequencies of the wild-type and 1-bp deletion sequences were similar in all tissues. We further confirmed that the mutation caused by the 1-bp deletion resulted in a stop codon.

**Figure 2:**
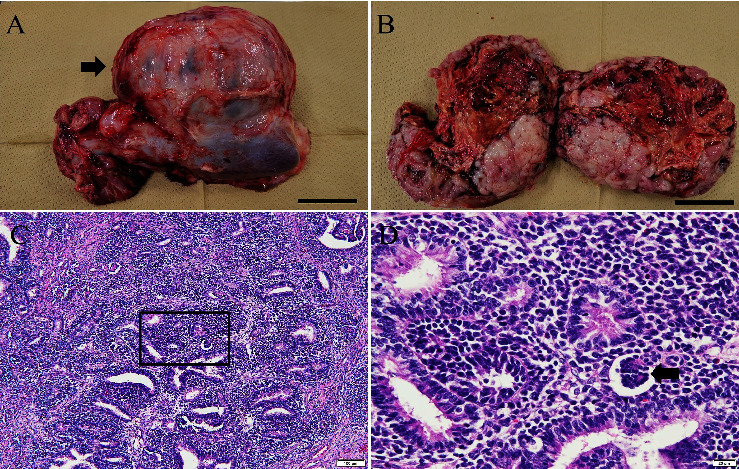
Gross and microscopic images of tumours detected in *TP53*-modified pig B. (a): Large nephroblastoma tumour mass (arrow) in the left kidney. (b): Cross-section of the tumour in the left kidney. Scale bar = 5 cm. (c): Neoplastic lesion composed of blastemal cells, epithelial cells that formed tubular and primitive glomerular-like structures, and stromal components. The predominant component is the blastema. 100x magnification. (d): Image within the square is shown in Figure 2(c). The blastemal cells are arranged in solid sheets. They are small round-to-oval cells with scant cytoplasm. Epithelial cells showing tubular patterns (arrowheads). A primitive glomerular-like structure (arrow). Each tumour cell has scant cytoplasm and oval nuclei with mild-to-moderate atypia. However, the mitotic index remained low. 400x magnification.

**Figure 3:**
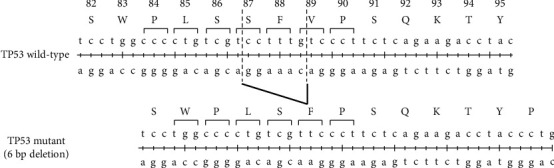
Alignment of nucleotides and amino acids of wild-type and mutant *TP53* (6-bp deletion) sequences. The human *TP53* sequence serves as the reference.

In pig B, tumours were found in both the kidneys but not in the other major organs. Gross examination revealed a pathognomonic lesion of a nephroblastoma-like tumour (Figures 2(a) and 2(b)). The solid tumour in the left kidney was located in the renal hilus (13 cm in diameter) and appeared as a solitary round multinodular mass. A solid mass (8 cm in diameter) was observed rostral to the right kidney. Microscopically, the neoplastic lesion consisted of blastema cells (small round-to-oval cells with scant cytoplasm) arranged in solid sheets, epithelial cells forming tubular and primitive glomerular-like structures, and stromal components (Figures 2(c) and 2(d)). Each tumour cell had a small amount of cytoplasm and oval nuclei with mild-to-moderate atypia. After gross and microscopic observation of the right and left renal masses, nephroblastoma was diagnosed. Sequence analysis revealed a mosaic genotype with 6- and 7-bp deletions in exon 3, and no wild-type sequences were present in any of the collected tissues (Figure 1). The frequency of the 6-bp deletion was higher than that of the 7-bp deletion in all tissues. In addition, the mutation caused by the 7-bp deletion resulted in a premature stop codon. We further analysed the positions and differences in amino acid modifications in the 6-bp deletion sequence compared with the *TP53* wild-type sequence of pigs (Q9TUB2.P53_PIG) and humans (P04637) from the UniProt database [8] using Clustal Omega alignment [9]. The truncated amino acid positions were located in the 87^th^ to 89^th^ amino acid sequence (Figure 3). Two amino acid deletions (serine (S) and phenylalanine (F)) and one substitution (valine (V) with F) were detected.

## 4. Discussion

TP53 is a tumour suppressor that promotes apoptosis following DNA damage. *TP53*-modified pigs have been extensively explored as human cancer models. In previous studies, pigs were used to model various *TP53* mutations, including biallelic or mosaic mutations, which resulted in the development of several types of tumours, such as lymphoma, nephroblastoma, osteosarcoma, and histiocytoma [3, 10]. In the present study, the tumour phenotypes of pig B with mosaic mutations included nephroblastoma. A survey of slaughtered pigs showed that the frequency of nephroblastoma is typically quite low [11]. Therefore, it remains unclear why the tumour was only found in the kidney and not in other organs. However, it is of interest why pigs with mosaic mutations show a tumour phenotype, while pigs carrying wild-type sequences do not develop tumours. To address this, we compared the genetic differences between the tumorigenic and nontumorigenic phenotypes of the two *TP53*-modified pigs.

In pig A, which had a mosaic genotype with the wild-type *TP53* sequence, genotyping analysis revealed a similar frequency of wild-type and 1-bp deleted sequences in all tissues. We further confirmed that the 1-bp deletion resulted in a premature stop codon but not a TP53 alteration. We speculated that the presence of the wild-type *TP53* sequence could rescue the tumour phenotype triggered by small deletions that do not result in TP53 protein alteration.

In pig B, we found an increased frequency of a 6-bp deletion genotype in all tissues. We hypothesized that a 6-bp deletion might contribute to the tumour phenotype. Deletion of a 6-bp sequence from the *TP53* sequence resulted in the absence of two amino acids at positions 87–89, and we further analysed the role of the truncated amino acids in tumour formation. Using the UniProt database [8], we found that the TP53 amino acid sequence from positions 1–313 interacted with cell cycle and apoptosis regulator 2 (CCAR2). CCAR2, also known as deleted in breast cancer 1 (DBC1), plays an important role in tumour suppression by regulating p53 [12]. Therefore, alterations in TP53 observed in our study may affect the function of DBC1 in regulating tumour suppression. Furthermore, the amino acid sequence of TP53 from positions 63 to 102 interacts with WWOX, a tumour suppressor that contains a WW domain and exhibits oxidoreductase activity. Upon activation, TP53 binds to WWOX, which in turn inhibits cell migration, thereby preventing metastasis and suppressing tumour formation [13]. Truncated amino acids within the same sequence range (63–102) could compromise the ability of WWOX to control metastasis. Collectively, our results suggest that TP53 mutations with truncated amino acids are associated with tumour formation.

In conclusion, no tumour phenotype was observed in the *TP53*-mosaic mutant pig A with the wild-type *TP53* sequence and a premature stop codon caused by a 1-bp deletion. In contrast, pig B, lacking the wild-type *TP53* sequence, exhibited nephroblastoma, possibly caused by the deletion of two amino acids in the TP53 sequence that controls tumour formation. In this study, a limited number of animals were used; however, our observations indicated that the absence of these amino acids may have triggered tumour formation.

## Figures and Tables

**Table 1:** Sequences of the TP53 target region obtained following the analysis of genomic DNA of ear biopsies from pigs A and B.

Pig	Gender	Genotype		Sequence	Indels	Frequency (%)	Total mutation rate (%)
			Target	GTA**GGTCTTCTGAGAAGGGACAA ***AGG*ACGACAGGGGCCAG			

A	♂	Mosaic		GTAGGTCTTCTGAGAAGGGACAAAGGACGACAGGGGCCAG	Wt	18875/44723 (42.2%)	24389/44723 (54.5%)
				GTAGGTCTTCTGAGAAGGG-CAAAGGACGACAGGGGCCAG	−1 bp	18780/44723 (42.0%)	
				GTAGGTCTTCTGAGAAGGGAGACCTACAAAGGACGACAGGGGCCAG	+6 bp	2833/44723 (6.3%)	
				GTAGGTCTTCTGAGAA—GACGACAGGGGCCAG	−9 bp	2776/44723 (6.2%)	

B	♂	Biallelic		GTAGGTCTTCTGAGAAG—GGACGACAGGGGCCAG	−7 bp	24942/50232 (49.7%)	48895/50232 (97.3%)
		(With in frame)		GTAGGTCTTCTGAGAAGGGA—ACGACAGGGGCCAG	−6 bp	23953/50232 (47.7%)	

The nucleotides in bold and italic font represent target sequences and PAM sequences of sgRNA, respectively. The nucleotides in underline represent the inserted sequence. Frequency was determined by deep sequencing analysis. Wt: wild-type.

## Data Availability

The data used to support the findings of this study are included within the article.

## Supplementary Materials

Supplementary Materials Supplementary Figure 1: genomic structure of the TP53 locus and sgRNA sequence targeting TP53 exon 3. The cutting site of sgRNA is represented as scissors along with a dotted line. Table S1: off-target analysis of the delivered piglets by deep sequencing. Table S2: frequency of the WT sequence at possible off-target sites.

## References

[1] Szymanska K., Hainaut P. (2003). TP53 and mutations in human cancer. *Acta Biochimica Polonica*.

[2] Donehower L. A., Soussi T., Korkut A. (2019). Integrated analysis of TP53 gene and pathway alterations in the cancer genome atlas. *Cell Reports*.

[3] Tanihara F., Hirata M., Nguyen N. T. (2018). Generation of a TP53-modified porcine cancer model by CRISPR/Cas9-mediated gene modification in porcine zygotes via electroporation. *PLoS One*.

[4] Gaj T., Gersbach C. A., Barbas C. F. (2013). ZFN, TALEN, and CRISPR/Cas-based methods for genome engineering. *Trends in Biotechnology*.

[5] Tanihara F., Hirata M., Nguyen N. T. (2020). Efficient generation of GGTA1-deficient pigs by electroporation of the CRISPR/Cas9 system into in vitro-fertilized zygotes. *BMC Biotechnology*.

[6] Brinkman E. K., Chen T., Amendola M., van Steensel B. (2014). Easy quantitative assessment of genome editing by sequence trace decomposition. *Nucleic Acids Research*.

[7] Glenn T. C. (2011). Field guide to next-generation DNA sequencers. *Molecular Ecology Resources*.

[8] The UniProt Consortium (2016). UniProt: the universal protein knowledgebase. *Nucleic Acids Research*.

[9] Sievers F., Wilm A., Dineen D. (2011). Fast, scalable generation of high-quality protein multiple sequence alignments using Clustal Omega. *Molecular Systems Biology*.

[10] Sieren J. C., Meyerholz D. K., Wang X. J. (2014). Development and translational imaging of a TP53 porcine tumorigenesis model. *Journal of Clinical Investigation*.

[11] Sandison A. T., Anderson L. J. (1968). Tumors of the kidney in cattle, sheep and pigs. *Cancer*.

[12] Qin B., Minter-Dykhouse K., Yu J. (2015). DBC1 functions as a tumor suppressor by regulating p53 stability. *Cell Reports*.

[13] Chou P.-Y., Lin S.-R., Lee M.-H., Schultz L., Sze C. I., Chang N. S. (2019). A p53/TIAF1/WWOX triad exerts cancer suppression but may cause brain protein aggregation due to p53/WWOX functional antagonism. *Cell Communication and Signaling*.

