# Supplementary material for: Mosaic TP53 Mutation on Tumour Development in Pigs: A Case Study
Source: Vet Med Int. 2023 Aug 14;2023:7000858. doi: 10.1155/2023/7000858 (PMC10442180; doi:10.1155/2023/7000858)

Supplementary1 Figure. Genomic structure of the TP53 locus and sgRNA sequence targeting TP53 exon 3. The cutting site of sgRNA is represented as scissors along with a dotted line.


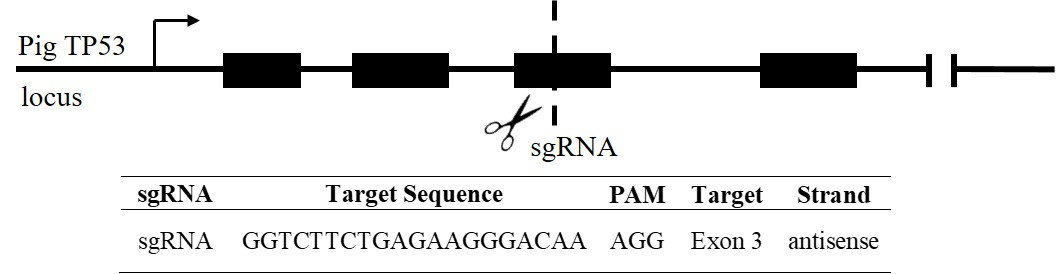

Supplement: Supplementary Materials — Supplementary Figure 1: genomic structure of the TP53 locus and sgRNA sequence targeting TP53 exon 3. The cutting site of sgRNA is represented as scissors along with a dotted line. Table S1: off-target analysis of the delivered piglets by deep sequencing. Table S2: frequency of the WT sequence at possible off-target sites. [file 7000858.f1.zip › Supplementary Figure.docx]
