# Supplementary material for: Mosaic TP53 Mutation on Tumour Development in Pigs: A Case Study
Source: Vet Med Int. 2023 Aug 14;2023:7000858. doi: 10.1155/2023/7000858 (PMC10442180; doi:10.1155/2023/7000858)
Supplement: Supplementary Materials — Supplementary Figure 1: genomic structure of the TP53 locus and sgRNA sequence targeting TP53 exon 3. The cutting site of sgRNA is represented as scissors along with a dotted line. Table S1: off-target analysis of the delivered piglets by deep sequencing. Table S2: frequency of the WT sequence at possible off-target sites. [file 7000858.f1.zip › Supplement_Table.docx]

S1 Table Off-target analysis of the delivered piglets by deep sequencing

| Off-target  Candidate |  | Genome sequence | Mismatch | Locus (strand) |
| --- | --- | --- | --- | --- |
|  | gRNA target | CCTTTGTCCCTTCTCAGAAGACCta | mismatch | locus (strand) |
| OT1 |  | CCATTGTCCCTTCTTAGAAGCCCxx | 2bp | Chr2:97623468-97623490 |
| OT2 |  | CCCTTGTCCCTTCTCAGGTGTCCxx | 3bp | Chr8:85524634-85524656 |
| OT3 |  | CCTTTGTCCCTTCCCGGAAGACCxx | 2bp | Chr9:3634099-3634121 |

Genome sequences and positions of possible off-target sites. Nucleotides in blue and red represent the target sequences and the PAM sequences of gRNA, respectively. Nucleotides in green represent mismatches with the gRNA sequence.

S2 Table Frequency of the WT sequence at possible off-target sites

| Off-target candidate | Pig | Reads aligned | unmodified reads | unmodified (%) |
| --- | --- | --- | --- | --- |
| OT1 | #3 | 45468 | 45132 | 99.26 |
|  | #4 | 67386 | 66946 | 99.35 |
| OT2 | #3 | 38348 | 38052 | 99.23 |
|  | #4 | 45424 | 45084 | 99.25 |
| OT3 | #3 | 48068 | 47749 | 99.34 |
|  | #4 | 47385 | 47059 | 99.31 |
